# Development of a Highly Sensitive FcMito qPCR Assay for the Quantification of the Toxigenic Fungal Plant Pathogen *Fusarium culmorum*

**DOI:** 10.3390/toxins10050211

**Published:** 2018-05-21

**Authors:** Katarzyna Bilska, Tomasz Kulik, Anna Ostrowska-Kołodziejczak, Maciej Buśko, Matias Pasquali, Marco Beyer, Anna Baturo-Cieśniewska, Marcin Juda, Dariusz Załuski, Kinga Treder, Joerg Denekas, Juliusz Perkowski

**Affiliations:** 1 Department of Microbiology and Mycology, University of Warmia and Mazury in Olsztyn, Oczapowskiego 1A, 10-719 Olsztyn, Poland; tomaszkulik76@gmail.com; 2 Department of Chemistry, Poznań University of Life Sciences, Wojska Polskiego 75, 60-625 Poznań, Poland; ostrowska.anna.maria@gmail.com (A.O.-K.); mabu@up.poznan.pl (M.B.); julperk@up.poznan.pl (J.P.); 3 Department of Food, Environmental and Nutritional Sciences (DEFENS), University of Milan, via Celoria 2, 20133 Milano, Italy; matias.pasquali@unimi.it; 4 Department Environmental Research and Innovation, Luxembourg Institute of Science and Technology, 41, rue du Brill, L-4422 Belvaux, Luxembourg; marco.beyer@list.lu; 5 Faculty of Agriculture and Biotechnology, Department of Phytopathology and Molecular Mycology, University of Technology and Life Sciences, Kordeckiego St. 20, 85-225 Bydgoszcz, Poland; baturo-a@utp.edu.pl (A.B.-C.); mar.bydg@gmail.com (M.J.); 6 Department of Plant Breeding and Seed Production, University of Warmia and Mazury in Olsztyn, Plac Łódzki 3, 10-727 Olsztyn, Poland; dariusz.zaluski@uwm.edu.pl; 7 Department of Agroecosystems, University of Warmia and Mazury in Olsztyn, Plac Łódzki 3, 10-727 Olsztyn, Poland; kinga.treder@uwm.edu.pl; 8 Agravis Technik Heide-Altmark GmbH, Hansestrasse 30, 29525 Uelzen, Germany; joergdenekas@googlemail.com

**Keywords:** *Fusarium culmorum*, qPCR assay, quantification, detection, mitochondrial DNA (mtDNA), Fusarium head blight (FHB)

## Abstract

*Fusarium culmorum* is a ubiquitous, soil-borne fungus (ascomycete) causing foot and root rot and Fusarium head blight on cereals. It is responsible for yield and quality losses as well as grain contamination with mycotoxins, which are a potential health hazard. An extremely sensitive mitochondrial-based qPCR assay (FcMito qPCR) for quantification of *F. culmorum* was developed in this study. To provide specificity, the FcMito assay was successfully validated against 85 *F. culmorum* strains and 53 isolates of 30 other fungal species. The assay efficiency and sensitivity were evaluated against different *F. culmorum* strains with various amounts of pure fungal DNA and in the presence of background wheat DNA. The results demonstrated the high efficiency of the assay (97.2–106.0%, R^2^-values > 0.99). It was also shown that, in the presence of background DNA, 0.01 pg of fungal template could be reliably quantified. The FcMito assay was used to quantify *F. culmorum* DNA using 108 grain samples with different trichothecene levels. A significant positive correlation was found between fungal DNA quantity and the total trichothecene content. The obtained results showed that the sensitivity of the FcMito assay was much higher than the nuclear-based qPCR assay for *F. culmorum*.

## 1. Introduction

A common soil-borne fungus, *Fusarium culmorum*, remains an important cereal pathogen of a wide range of small-grain cereals, as well as maize [1,2]. On wheat and barley, it causes two distinct diseases: FRR (Fusarium foot and root rot) and FHB (Fusarium head blight), also known as ear blight or scab [1]. Both diseases cause significant yield losses and the latter results in contamination of the grain with trichothecenes, which may pose a grave threat to both food and feed safety [3,4,5,6]. Trichothecenes produced by *F. culmorum* include deoxynivalenol (DON), nivalenol (NIV), and their acetylated derivatives, 3-acetylodeoxynivalenol (3ADON) and 4-acetylnivalenol (4ANIV, syn. fusarenon X) [7], which differ in toxicity to mammals and plants [8]. Options of chemical control are very limited. *F. culmorum* can be controlled by fungicides containing triazoles as active ingredients, while strobilurins such as trifloxystrobin and succinate dehydrogenase inhibitors such as isopyrazam are hardly effective [9].

Before 2000, *F. culmorum* was the predominant agent of FHB in Northern, Central, and Western Europe [1,10]. However, since that time, a change has been observed in many European countries in the distribution of *F. culmorum*, which appears to be replaced by *F. graminearum* sensu stricto (s.s.) [11]. This progressive switch may be explained by the increased production of maize, which favors the production of ascospores by *F. graminearum* s.s. Other causes of *F. culmorum* to *F. graminearum* s.s. replacement may be related to the gradual adaptation of *F. graminearum* s.s. to colder climates [12,13] or to the recorded rise in average temperatures caused by climate change [1,14,15,16]. It is, however, noteworthy that *F. culmorum* can predominate in certain European locations. For example, in Luxembourg, following the dry year 2011, *F. culmorum* was identified from the majority of symptomatic wheat heads, whereas only 10% were infected by *F. graminearum* s.s. [17]. *F. culmorum* is now frequently reported as the main agent of FRR and FHB in the Mediterranean region, which is contrary to surveys from Central and Northern Europe [1,18,19,20,21,22,23,24,25].

Traditionally, identification of *F. culmorum* is based on the shape of the macroconidia formed in sporodochia [1,26]. However, morphological identification of Fusaria is time-consuming, requires qualified experts in the field of culturing fungi [27], and precludes the distinction of species frequently occurring on wheat [26]. In addition, ascomycetes cannot be reliably quantified by the viable count procedures [28,29]. Recently, quantitative polymerase chain reaction (qPCR) has been found to be a most promising method for fungal quantification from different environmental samples. qPCR offers several advantages over other diagnostic techniques, including higher sensitivity and specificity and a rapid turnaround time. At least four specific real-time PCR assays have previously been designed for *F. culmorum*, using either SybrGreen or TaqMan chemistries [30,31,32,33]. However, fungal quantification is sometimes a challenge, mostly due to the relatively low fungal biomass in environmental samples and the fungal cell wall structure itself, which makes nucleic acid extraction difficult [34]. The sensitivity of qPCR assays can be improved with diagnostic assays focusing on various multi-copy DNA regions, such as mitochondrial DNA (mtDNA). As was successfully demonstrated with the development of the FgMito assay for quantification of *F. graminearum* s.s. [35].

The present study sought to design a highly sensitive FcMito qPCR assay to quantify *F. culmorum*. To ensure high assay sensitivity, primers and a minor-groove binding (MGB) probe were prepared based on multi-copy mitochondrial DNA. The specificity of the assay was evaluated against both a broad range of *F. culmorum* strains from distinct localities as well as other *Fusarium* species. The assay’s efficiency and sensitivity were further evaluated against a test panel of different *F. culmorum* strains with various amounts of pure fungal DNA as well as in the presence of wheat background DNA. *F. culmorum* was quantified using the FcMito assay from 108 field samples with different trichothecene amounts.

## 2. Results

### 2.1. The Design of a Primer/Probe Set for Specific Quantification of F. culmorum Based on the Mitochondrial COX2 Gene

To design a primer/probe set specific for *F. culmorum*, complete mitogenomes of *F. culmorum*, *F. graminearum* s.s., and *F. gerlachii* were aligned. An intron3 within the *COX2* gene was present in *F. culmorum* only, based on which a primer/probe set was designed for specific quantification of this species. In silico specificity of primers and the designed probe was further confirmed by BLAST searches.

### 2.2. Optimizing a TaqMan Assay Specific for F. culmorum

The 138 *Fusarium* strains were used to evaluate the specificity of the FcMito assay (Table S1). All 85 *F. culmorum* strains emitted a fluorescent signal with the FcMito assay, whereas no amplification was noted on the remaining tested non-target Fusaria. The efficiency of the FcMito assay ranged from 98.5 to 100.5%, while the coefficient of determination was greater than R^2^ = 0.995 (Table 1). No significant differences were recorded in the C_T_ range, R^2^, or efficiency between pure strain standards or standards mixed with 30 ng of wheat background DNA. No false-positive signals were detected when using 30 ng of wheat DNA (Table 2). Six low concentrations of *F. culmorum* DNA (2, 0.5, 0.2, 0.05, 0.02, and 0.01 pg) showed no false-negative results in the presence of background wheat DNA. A false-negative rate of 34.7% was found for the lowest DNA input (0.005 pg). In this way, the limit of quantification (LOQ) of the FcMito assay could be established as 0.05 pg.

### 2.3. The Quantification of F. culmorum DNA from Naturally Contaminated Grain Samples

The FcMito assay was used to quantify *F. culmorum* DNA from 108 grain samples (Table S2). The presence of *F. culmorum* was revealed in all of them. The obtained C_T_ values ranged from 21.46 to 36.94, while the estimated fungal DNA amount differed significantly between samples, ranging from 61.969 to 0.002 pg. However, most of the grain samples (*n* = 78) contained a very low amount of *F. culmorum* DNA (<1 pg). Grain samples were additionally examined with the real-time PCR assay, which targeted the nuclear genome of *F. culmorum* [30]. Negative results were obtained for 60 samples. All of these samples contained very low amounts of *F. culmorum*, ranging from 1.753 to 0.002 pg, as quantified with the FcMito assay. Thus, over half of the amount of the samples analyzed with a nuclear-based assay produced false-negative results.

The DNA from *F. graminearum* s.s. was found in almost half of the samples (*n* = 53). Interestingly, in most samples exhibiting increased levels of *F. graminearum* s.s., a very low quantity of *F. culmorum* was revealed.

The grain samples were then analyzed for the presence of trichothecenes. Deoxynivalenol was the dominant trichothecene compound (Table S2). Mycotoxins were found in all samples except for two. To determine the existence of the relationship in the total trichothecenes and the DNA quantity in grain samples, Spearman’s rank correlation was calculated separately for *F. culmorum*, *F. graminearum* s.s., and both species together. Spearman’s rank correlation was used because non-normal distribution of the data. In all three cases, statistical analysis revealed positive correlations between the fungal DNA and the total trichothecene content (*r* = 0.36, *r* = 0.35, *r* = 0.49, accordingly, *p* < 0.001).

## 3. Discussion

There are two major *Fusarium* species responsible for FHB in Europe: *F. culmorum* and *F. graminearum* s.s. Reliable identification and quantification of these species forms the basis of all studies related to population dynamics, fungal ecology, toxicology, and the efficacy of crop protection measures. Nowadays, qPCR is one of the most promising tools for quantification of Fusaria from environmental samples. However, the quantification of fungal DNA can often be hampered by the relatively low levels of fungal loads in environmental samples and the structure of the fungal cell wall—making disruption for nucleic acid extraction difficult [34]. It is possible to improve the sensitivity of fungal quantification by diagnostic assays targeting mtDNA. Considering the relatively small differences for mean and median of quantified DNA for 0.01 and 0.02 pg (Table 2), the limit of quantification (LOQ) of the FcMito assay was determined as 0.05 pg. This is 18-fold lower than LOQ of another *F. culmorum* specific TaqMan assay targeting the nuclear genome [30]. It was shown that the FcMito assay could detect 0.005 pg of the input template, but only with a 35% false-negative rate. Thus, the limit of detection (LOD) of the designed assay could be determined between 0.05 and 0.005 pg. The haploid cell of *F. culmorum* contains 42 Mb [36], which equals 0.04 pg. Therefore, LOQ of the FcMito assay (0.05 pg) is equal to approximately one and a quarter of the haploid cell of *F. culmorum*. A comparison of the LOQ values of both FcMito and FgMito assays shows a 4-fold lower LOQ of FcMito than the FgMito assay. This can be explained by incorporation of a primer with an additional mismatch in the 3rd last nucleotide from the 3′ end of the FgMito assay. Although this modification increased the specificity of the assay [35], it negatively affected the LOQ and LOD of the FgMito assay (data not shown).

Atoui et al. [37] claimed that the best way to identify grain contamination by fungi is targeting the mycotoxigenic genes specifically where a particular mycotoxin can be produced by a number of species. However, it is occasionally impossible to distinguish a particular species. Atoui et al. [37] found that primers targeting the PKS13 gene involved in zearalenone (ZEA) biosynthesis detected *F. graminearum* s.s. and *F. culmorum*. A similar assay was developed by Meng et al. [38] and was specific for the zearalenone-producing *F. graminearum* s.s., *F. culmorum*, and *F. cerealis*. FcMito assay is species-specific, as it only amplifies the *F. culmorum* DNA (Table S1).

In this study, high efficiency (98.5–100.5%) and high coefficients of determination (over R^2^ = 0.995) were acquired for a series dilution of pure strain standards. Moreover, there were no significant differences in amplification profiles between pure strain standards and standards mixed with 30 ng of wheat background DNA. This indicates that, since wheat DNA had no impact on FcMito assay, it can be successfully used in field sample analysis.

This study found a positive correlation between the *F. culmorum* DNA and the total trichothecenes present in naturally contaminated grains. Despite Spearman’s rank correlation coefficient (r = 0.34) not being high, it was still significant. Similar results were obtained when correlating quantities of *F. graminearum* s.s. DNA to trichothecenes [35]. However, we found that correlation of the sum of trichothecenes to the sum of *F. culmorum* and *F. graminearum* s.s. DNA resulted in an increase in Spearman’s rank correlation coefficient (r = 0.49), which indicates that a more reliable prediction of trichothecene content in the grain requires quantification of both species. This also confirmed that both *F. culmorum* and *F. graminearum* s.s. are responsible for the current contamination of European grains with type B trichothecenes [24].

Fusaria differ significantly in pathogenicity, toxigenicity, and sensitivity to commonly used fungicides. Identification and quantification of predominant species is important in preventing and controlling the FHB epidemic. Furthermore, the quantification of fungal DNA can indicate plausible mycotoxin contamination. This may help in the prediction of potential toxicological risks. Due to its extremely high sensitivity, a FcMito assay may be used to study infection strategy of the pathogen in biomass-limited samples such as individual floral organs of cereals. Together with other mitochondrial-based assays, the assay developed in this study could be applied to reveal tissue-specific infection patterns in naturally infected caryopses, paleas, lemmas, and glumes on which compound appressoria are formed at initial stages of the infection process [39]. Our assay may also be used to characterize fungal concentrations in environmental samples with relatively lower amounts of fungal biomass such as air dust, soil, and water, without the need to cultivate fungi on artificial media. Finally, the mitochondrially based assay developed in this study would be valuable in many plant-breeding programs, as well as an initial step in food and feed quality assessment.

## 4. Materials and Methods

### 4.1. Fungal Strains

Table S1 lists the 138 *Fusarium* strains used in the specificity test. Polish isolates of *F. culmorum* [40] are kept in the Department of Phytopathology and Molecular Mycology of the University of Technology and Life Sciences (Bydgoszcz, Poland). The DBNP strains are located in the Department of Microbiology and Mycology of the University of Warmia and Mazury in Olsztyn (Olsztyn, Poland). The tested CBS strains are held in the Westerdijk Fungal Biodiversity Institute (Utrecht, The Netherlands). The strains from Italy and Luxemburg are kept in the Fungal Culture Collection of the Department Environmental Research and Innovation of the Luxembourg Institute of Science and Technology and are accessible at www.luxmcc.lu [41]. The *Fusarium* isolates were maintained at 25 °C on a potato dextrose agar (PDA) before DNA extraction was conducted.

### 4.2. Grain Samples

The 108 grain samples were analyzed in the study presented in this paper (Table S2). The wheat samples from Luxembourg (n = 13) (received from the Luxembourg Institute of Science and Technology) were collected in 2007 and 2008 [42]. Polish samples were harvested in 2011 and 2012. An IKA A10 analytical grinding mill (IKA Werke GmbH & Co. KG, Staufen im Breisgau, Germany) was used to initially grind 100 g of grain from each sample. Samples were kept at −25 °C.

### 4.3. DNA Extraction

The DNA extraction involved 0.1 g of mycelium collected from the top of PDA plates as well as 0.25 g of ground grain per sample. These samples were homogenized twice (30 s at a speed of 6.0 m/s) using a FastPrep-24 instrument (MP Biomedicals, Solon, OH, USA). A ChargeSwitch^®^ gDNA Plant Kit (Invitrogen, Carlsbad, CA, USA) was used to isolate genomic DNA following the manufacturer’s protocol. A Qubit^®^ 2.0 Fluorometer and a Qubit^®^dsDNA BR Assay (Invitrogen, Carlsbad, CA, USA) were used to determine fungal and plant DNA concentrations according to the manufacturer’s recommendations.

### 4.4. Design of a Primer/Probe Set Specific for F. culmorum

To find the polymorphic site to design a primer/probe set specific for *F. culmorum*, complete mitogenomes of *F. culmorum* (GenBank: NC_026993.1), *F. graminearum* s.s. (GenBank: NC_009493.1, DQ364632.1, KR011238.1, KP966561.1, KP966560.1, KP966559.1, KP966558.1, KP966557.1, KP966556.1, KP966555.1, KP966554.1, KP966553.1, KP966552.1, KP966551.1, KP966550.1) and *F. gerlachii* (GenBank: KM486533) were aligned. The sequence analysis revealed that the *COX2* intron3 (3358 bp, 34.300–37.658) was present in *F. culmorum* only, which was then used to design primers and probe (Table 3) using the PRIMER EXPRESS 3.0 software package (Applied Biosystems, Foster City, CA, USA). The probe included a minor groove binder (MGB) moiety at the 3′ was labeled with 6-carboxyfluorescein (FAM) at the 5′-end. The probes were obtained from the ABI PRISM Primers and TaqMan Probe Synthesis Service and the primers were synthesized by Sigma-Aldrich (St. Louis, MO, USA).

### 4.5. Optimization of a TaqMan Assay Specific for F. culmorum

The TaqMan reaction was performed according to Kulik et al. [35] in a 7500 Fast Real-Time PCR System (Applied Biosystems, Carlsbad, CA, USA). The optimized TaqMan assay specificity was verified by testing a total of 138 fungal strains (Table S1). The efficiency and sensitivity of the assay were determined using a ten-fold serial dilution of various amounts of gDNA (in picograms) of six *F. culmorum* strains as a template (Table 1). The background DNA impact was considered by testing the efficiency and sensitivity of the assay in the presence of 30 ng of wheat DNA. Four replicates of each dilution were prepared for each TaqMan experiment. Two non-template controls (NTCs) were used: water-only and background DNA-only samples. The reaction efficiency and coefficient of determination (R^2^) were calculated with the obtained C_T_-values. The TaqMan reaction was performed in 96 replicates, using 30 ng of wheat DNA as a template in order to verify the false-positive rate. To identify false-negative results, seven low concentrations of *F. culmorum* DNA (2, 0.5, 0.2, 0.05, 0.02, 0.01, and 0.005 pg) were analyzed in the presence of 30 ng of wheat DNA (Table 2). The concentration of each template was then analyzed in 72 replicates.

### 4.6. Quantification of F. culmorum DNA from Naturally Contaminated Grain Samples

Plant Internal Positive Control (pIPC) was used for normalization the DNA samples extracted from grains, according to Kulik et al. [43]. Subsequently, all samples were analyzed for the presence and quantity of *F. culmorum* using the FcMito assay. The amount of *F. culmorum* DNA (pg) was evaluated from C_T_ values using a standard curve. All template concentrations were analyzed in three replicates under the conditions described previously. The obtained results were compared with the results of *F. culmorum* and *F. graminearum* s.s. quantification from a previous study by Kulik et al. [35]. Differences in sensitivity between the FcMito assay and nuclear-based assay [30] were revealed by comparing the C_T_ values of both assays.

### 4.7. Trichothecene Determination from the Grain Samples

The levels of DON, 3ADON, 15ADON, and NIV were determined in grain samples. The Polish samples were analyzed for trichothecene presence according to the protocol described by Perkowski et al. [44], while samples from Luxembourg were analyzed according to Giraud et al. [42].

### 4.8. Statistical Analyses

Statistical analyses were conducted using STATISTICA (Data Analysis Software System, ver. 12.5; StatSoft Inc., Tulsa, OK, USA, 2014). The relationship between the DNA quantity and the total trichothecene content in grain samples (*p* < 0.05) was established by Spearman’s rank correlation. The mean (pg) and median (IQR) (pg) quantities of seven low concentrations of the template (2, 0.5, 0.2, 0.05, 0.02, 0.01, and 0.005 pg) analyzed in the presence of background wheat DNA were determined.

## Figures and Tables

**Table 1:** Validation results of FcMito assay based on 10 PCR runs using pure strain standards and templates mixed with 30 ng of wheat DNA.

Strain	Assay Quantitative Dynamic Range (pg) ^a^	C_T_ Range	R^2^	Efficiency (%)
M601	6840–0.68	15.31 ± 0.17–28.18 ± 0.20	0.998	99.2
ZFc 0502	6500–0.65	14.99 ± 0.19–28.15 ± 0.17	0.998	99.9
ZFc 0601	27,320–2.73	12.72 ± 0.18–25.98 ± 0.16	0.998	99.0
ZFc 0601 ^b^		12.9 ± 0.09–26.17 ± 0.12	0.999	98.9
CBS 110568	8720–0.67	23.34 ± 0.12–36.68 ± 0.13	0.998	99.7
CBS 110568 ^b^		22.98 ± 0.14–35.85 ± 0.96	0.999	98.1
CBS 171.28	5440–0.54	23.79 ± 0.12–36.55 ± 0.14	0.995	100.5
CBS 171.28 ^b^		23.86 ± 0.10–36.22 ± 0.06	0.997	106.0
MCR 320	2620–0.26	15.89 ± 0.09–29.28 ± 0.21	0.999	98.5
MCR 320 ^b^		15.88 ± 0.07–29.45 ± 0.13	0.999	97.2

^a^ Fungal DNA was quantified by Qubit fluorometer in three independent measurements; ^b^ Diluted in the presence of 30 ng of background wheat DNA.

**Table 2:** Results of the quantity median (IQR) and quantity mean of seven low concentrations of *F. culmorum* DNA (in picograms) analyzed in the presence of 30 ng of wheat DNA.

Amount of Input Template (pg)	No. of Positive Amplifications	Quantity Mean (pg)	Quantity Median (IQR) (pg)
2	72/72	2.218 (1.821–2.94)	2.191
0.5	72/72	0.489 (0.39–0.62)	0.483
0.2	72/72	0.228 (0.161–0.578)	0.22
0.05	72/72	0.053 (0.031–0.11)	0.052
0.02	72/72	0.014 (0.001–0.064)	0.011
0.01	72/72	0.011 (0.002–0.035)	0.010
0.005	47/72	0.008 (0.001–0.084)	0.004
30 ng of wheat background DNA only	0/96	-	-
No template	0/96	-	-

**Table 3:** Primers and MGB probe developed for quantification of *F. culmorum*.

Primer/Probe Name	Primer/TagMan Probe Sequence
COX2_1	TCGTTGACGGTGAGGGTTGT
COX2_2	GACTCGAACACGTCAACCAACTT
COX2 probe	FAM-CGGTTATTATTTCGAAAAGT-MGB

## Supplementary Materials

The following are available online at http://www.mdpi.com/2072-6651/10/5/211/s1. Table S1: List of fungal strains used for specificity testing of FcMito qPCR assay. Table S2: Results of quantification of *F. culmorum* and *F. graminearum* s.s. DNA from cereals with defined levels of trichothecenes using three different TaqMan assays.
