# Supplementary material for: Development of a Highly Sensitive FcMito qPCR Assay for the Quantification of the Toxigenic Fungal Plant Pathogen Fusarium culmorum
Source: Toxins (Basel). 2018 May 21;10(5):211. doi: 10.3390/toxins10050211 (PMC5983267; doi:10.3390/toxins10050211)
Supplement: Supplementary file 1 [file toxins-10-00211-s001.pdf]

Supplementary Materials: Development of a Highly Sensitive FcMito qPCR Assay for the Quantification of the Toxigenic Fungal Plant Pathogen *Fusarium culmorum*

Katarzyna Bilska, Tomasz Kulik, Anna Ostrowska-Kołodziejczak, Maciej Buśko, Matias Pasquali, Marco Beyer, Anna Baturo-Cieśniewska, Marcin Juda, Dariusz Załuski, Kinga Treder, Joerg Denekas, and Juliusz Perkowski

Table S1. List of fungal strains used for specificity testing of FcMito qPCR assay.

Fungal species	Strain code	Geographical origin, host/habitat of origin	FcMito qPCR assay
F. culmorum	CBS 110568	France, wheat	+
	CBS 139512	Poland, wheat	+
	CBS 129.73	Portugal, *Populus nigra*	+
	CBS 110269	Canada	+
	CBS 251.52	*Triticum aestivum*, grain, cv. Koga	+
	CBS 122.73	United Kingdom, *Triticum*	+
	CBS 256.51	Netherlands, soil	+
	CBS 171.28	Unknown	+
	CBS 579.97	Denmark, chopped stems, buried in soil	+
	CBS 173.31	Canada, *Avena sativa*	+
	MCR331	Italy, Sardinia	+
	MCR320	Italy, Sardinia	+
	MCR366	Italy, Sardinia	+
	MCR 321	Italy, Sardinia	+
	M601	Luxembourg, Burmerange	+
	M233	Luxembourg, Reisdorf	+
	Fc1g	Germany, winter wheat	+
	Fc2g	Germany, winter wheat	+
	Z Fc 0502*	Poland, potato	+
	Z Fc 0503*	Poland, potato	+
	Z Fc 0505*	Poland, potato	+
	Z Fc 0506*	Poland, potato	+
	Z Fc 0601*	Poland, potato	+
	Z Fc 0602*	Poland, potato	+
	Z Fc 0603*	Poland, potato	+
	Z Fc 0606*	Poland, potato	+
	Z Fc 0608*	Poland, potato	+

	Z Fc 0706*	Poland, potato	+
	Po Fc 0601*	Poland, winter wheat	+
	Po Fc 0602*	Poland, winter wheat	+
	Po Fc 0603*	Poland, winter wheat	+
	Po Fc0604*	Poland, winter wheat	+
	Po Fc 0605*	Poland, winter wheat	+
	Po Fc 0606*	Poland, winter wheat	+
	Po Fc 0607*	Poland, winter wheat	+
	Po Fc 0608*	Poland, winter wheat	+
	Po Fc 0610*	Poland, winter wheat	+
	Po Fc 0611*	Poland, winter wheat	+
	Po Fc 0701*	Poland, winter wheat	+
	Po Fc 0702*	Poland, winter wheat	+
	Po Fc 0703*	Poland, winter wheat	+
	Po Fc 0704*	Poland, winter wheat	+
	Po Fc 0705*	Poland, winter wheat	+
	Po Fc 0707*	Poland, winter wheat	+
	Po Fc 0708*	Poland, winter wheat	+
	Po Fc 0710*	Poland, winter wheat	+
	Po Fc 0711*	Poland, winter wheat	+
	Po Fc 0712*	Poland, winter wheat	+
	Po Fc 0713*	Poland, winter wheat	+
	Po Fc 0714*	Poland, winter wheat	+
	Po Fc 0715*	Poland, winter wheat	+
	Pj Fc 0702*	Poland, spring wheat	+
	Pj Fc 0703*	Poland, spring wheat	+
	Pj Fc 0704*	Poland, spring wheat	+
	Pj Fc 0705*	Poland, spring wheat	+
	Pj Fc 0706*	Poland, spring wheat	+
	Pj Fc 0707*	Poland, spring wheat	+
	Pj Fc 0708*	Poland, spring wheat	+
	Pj Fc 0709*	Poland, spring wheat	+
	Pj Fc 0710*	Poland, spring wheat	+
	Pj Fc 0711*	Poland, spring wheat	+
	Pj Fc 0712*	Poland, spring wheat	+
	Pj Fc 0801*	Poland, spring wheat	+
	Pj Fc 0802*	Poland, spring wheat	+
	Pj Fc 0803*	Poland, spring wheat	+

	Jj Fc 0502*	Poland, spring barley	+
	Jj Fc 0504*	Poland, spring barley	+
	Jj Fc 0512*	Poland, spring barley	+
	Jj Fc 0518*	Poland, spring barley	+
	Jj Fc 0526*	Poland, spring barley	+
	Jj Fc 0621*	Poland, spring barley	+
	Jj Fc 0629*	Poland, spring barley	+
	Jj Fc 0640*	Poland, spring barley	+
	Jj Fc 0641*	Poland, spring barley	+
	Jj Fc 0649*	Poland, spring barley	+
	Jj Fc 0801*	Poland, spring barley	+
	Jj Fc 0802*	Poland, spring barley	+
	Pz Fc 0802*	Poland, triticale	+
	Pz Fc 0803*	Poland, triticale	+
	Pz Fc 0804*	Poland, triticale	+
	Zo Fc 0801*	Poland, winter rye	+
	Zo Fc 0802*	Poland, winter rye	+
	Zo Fc 0803*	Poland, winter rye	+
	Zo Fc 0804*	Poland, winter rye	+
	O Fc 0801*	Poland, oats	+
F. aethiopicum	CBS 123667	Ethiopia, Gugsa womberma district, wheat	-
F. acaciae-mearnsii	CBS 110253	South Africa, *Acaciae mearnsii* (Leguminosae-mimosoideae)	-
	CBS 123662	Australia, soil	-
F. asiaticum	CBS 110258	China, Shanghai Province, wheat	-
	CBS 110257	Japan, barley	-
	CBS 110256	barley	-
F. austroamericanum	CBS 110246	Brazil, maize	-
	CBS 110245	Venezuela, herbaceous vine	-
	CBS 110244	Brazil, polypore	-
F. boothii	CBS 110270	South Africa	-
	CBS 110250	South Africa, corn	-
F. brasilicum	CBS 119180	Brazil, oats	-
F. cortaderiae	CBS 123655	New Zealand, corn	-
F. gerlachii	CBS 123666	USA, North Dakota, wheat	-
F. graminearum s. s.	CBS 139513	Argentina, Tandil, barley	-
	CBS 128539	Belgium, wheat	-
	CBS 110263	Iran, maize	-
	CBS 389.62	Netherlands, wheat	-

	CBS 138561	Poland, Tywęzy, wheat	-
	CBS 138563	Poland, wheat	-
	CBS 119800	South Africa, corn	-
	CBS 123688	Sweden, oats	-
	CBS 110266	USA, Kansas, wheat	-
F. louisianense	CBS 127524	USA, Louisiana, wheat	-
F. meridionale	CBS 110247	New Caledonia, orange twig	-
	CBS 110248	Nepal, maize	-
	CBS 110249	South Africa, soil	-
F. mesoamericanum	CBS 415.86	Honduras, Musa (Musaceae)	-
F. nepalense	CBS 127503	Nepal, rice	-
F. ussurianum	CBS 123754	Russian Federation, near Ussuriysk	-
	CBS 123751	Russian Federation, Jewish Autonomous Region, wheat	-
F. vorosii	CBS 123664	Japan, wheat	-
Unknown	CBS 123663	South Africa, soil	-
F. cerealis	CBS 195.80	Colombia, burnt páramo soil	-
	CBS 623.85	Netherlands, potato tuber buried in soil	-
	CBS 314.73	New Zealand, Azalea	-
F. pseudograminearum	CBS 109956	Australia, barley	-
	CBS 109953	New South Wales, soil	-
F. avenaceum	DBNP 0404	Poland, wheat	-
	DBNP 03162	Poland, wheat	-
F. aywerte	CBS 395.96	Australia, soil under *Plectrachne sp.*	-
F. chlamydosporum	CBS 445.67	Australia, wheat	-
F. graminum	CBS 119845	Australia, *Claviceps paspali* sclerotia on *Paspalum sp.*	-
F. oxysporum	CBS 620.87	Denmark, barley	-
F. oxysporum f. sp. pisi	CBS 127.73	United Kingdom, *Pisum sativum*	-
F. poae	CBS 180.96	Norway, wheat	-
F. sambucinum var. sambucinum	CBS 135.73	Egypt, *Lycopersicon esculentum*	-
F. venenatum	CBS 458.93	Austria, Rohrau, wheat	-
F. tricinctum	CBS 410.86	Denmark, mouldy grain	-
	DBNP 168bm	Poland, wheat	-
F. verticillioides	CBS 734.97	Germany, maize	-
F. nurragi	CBS 393.96	Australia, soil	-
F. reticulatum	CBS 618.87	Denmark, soil from fruit plantation	-

* strains from Baturo-Cieśniewska and Suchorzyńska (2011).

Table S2. Results of quantification of *F. culmorum* and *F. graminearum* s.s. DNA from cereals with defined levels of trichothecenes using three different TaqMan assays.

Sample no.	Cereal crop	FcMito assay		Nuclear based assay specific for *F. culmorum* (Waalwijk et al 2004)		FgMito assay specific for *F. graminearum* s. s (Kulik et al 2015)		Trichothecene levels (µg/kg)			
		C_T Mean ± C_T SD	Mean quantity ± quantity SD pg	C_T Mean ± C_T SD	Mean quantity ± quantity SD pg	C_T Mean ± C_T SD	Mean quantity ± quantity SD pg	DON	3ADON	15ADON	NIV
1	wheat	23.24 ± 0.09	18.211 ± 1.13	29.61 ± 0.13	5.858 ± 0.534	25.39 ± 0.19	20.605 ± 2.742	681	nd	nd	nd
2	wheat	24.06 ± 0.08	10.437 ± 0.583	30.71 ± 0.1	2.746 ± 0.198	25.49 ± 0.06	19.177 ± 0.74	268	nd	nd	341
3	wheat	23.33 ± 0.25	17.261 ± 2.788	27.98 ± 0.06	18.008 ± 0.739	27.85 ± 0.05	3.716 ± 0.137	1903	nd	nd	nd
4	wheat	25.92 ± 0.1	2.908 ± 0.192	30.59 ± 0.1	2.977 ± 0.213	27.25 ± 0.06	5.641 ± 0.231	842	nd	nd	nd
5	wheat	25.5 ± 0.13	3.882 ± 0.35	31.03 ± 0.27	2.224 ± 0.386	28.04 ± 0.003	3.259 ± 0.006	968	nd	nd	532
6	wheat	24.47 ± 0.19	7.886 ± 1.015	29.62 ± 0.05	5.788 ± 0.184	27.76 ± 0.07	3.965 ± 0.187	489	nd	nd	202
7	wheat	21.46 ± 0.12	61.969 ± 5.019	26.99 ± 0.09	35.203 ± 2.105	22.5 ± 0.07	152.37 ± 7.907	1215	nd	nd	462
8	wheat	35.42 ± 2.67	0.012 ± 0.014	nd	-	nd	-	nd	nd	nd	nd
9	wheat	35.33 ± 0.74	0.008 ± 0.003	nd	-	22.81 ± 0.09	61.361 ± 3.952	333	nd	nd	nd
10	wheat	29.56 ± 0.03	0.381 ± 0.007	nd	-	nd	-	nd	nd	nd	nd
11	wheat	35.75 ± 2.62	0.012 ± 0.016	nd	-	23.44 ± 0.33	39.869 ± 9.133	34	1	1	1
12	wheat	31.83 ± 0.29	0.074 ± 0.015	nd	-	33.78 ± 0.43	0.027 ± 0.008	1	nd	0	1
13	wheat	36.87 ± 1.53	0.004 ± 0.003	nd	-	nd	-	2	nd	nd	nd
14	wheat	33.89 ± 0.81	0.02 ± 0.009	nd	-	35.34 ± 0.88	0.01 ± 0.005	1	nd	nd	nd
15	wheat	36.94 ± 0.06	0.002 ± <0.001	nd	-	24.23 ± 0.78	25.124 ± 12.344	6	1	nd	nd
16	crop mixture	26.24 ± 0.15	2.301 ± 0.231	31.54 ± 0.08	0.008 ± <0.001	nd	-	9	nd	nd	nd
17	rye	33.0 ± 1.44	0.033 ± 0.025	nd	-	nd	-	5	6	4	4
18	spelt	31.56 ± 0.3	0.088 ± 0.017	nd	-	33.96 ± 0.63	0.024 ± 0.012	23	nd	nd	nd
19	wheat	26.84 ± 0.13	2.017 ± 0.168	31.08 ± 0.1	0.142 ± 0.01	34.28 ± 1.32	0.023 ± 0.015	11	11	6	11
20	rye	34.88 ± 0.64	0.01 ± 0.004	nd	-	35.35 ± 0.81	0.009 ± 0.004	nd	nd	nd	14
21	crop mixture	34.95 ± 0.61	0.012 ± 0.004	nd	-	nd	-	13	nd	nd	8
22	spelt	34.32 ± 0.57	0.018 ± 0.006	nd	-	nd	-	11	nd	nd	nd
23	wheat	26.69 ± 0.05	2.429 ± 0.077	30.83 ± 0.2	0.236 ± 0.012	30.83 ± 1.43	0.27 ± 0.18	7	nd	nd	2
24	wheat	29.24 ± 0.11	0.324 ± 0.024	nd	-	nd	-	115	nd	nd	nd
25	oat	36.61 ± 1.27	0.003 ± 0.002	nd	-	nd	-	nd	nd	nd	9
26	rye	33.63 ± 0.38	0.015 ± 0.004	nd	-	nd	-	nd	nd	nd	9
27	crop mixture	27.52 ± 2.52	1.753 ± 2.083	nd	-	nd	-	3	1	3	nd
28	wheat	25.16 ± 0.16	4.844 ± 0.544	30.5 ± 0.1	0.079 ± 0.015	29.79 ± 0.59	0.468 ± 0.206	82	nd	nd	3
29	rye	28.22 ± 0.22	0.808 ± 0.122	32.21 ± 0.1	0.114 ± 0.005	28.97 ± 1.01	0.948 ± 0.595	8	nd	nd	9
30	rye	29.96 ± 0.25	0.297 ± 0.045	nd	-	nd	-	16	nd	nd	6
31	crop mixture	29.49 ± 0.22	0.347 ± 0.052	32.47 ± 0.22	0.004 ± 0.001	29.53 ± 1.11	0.627 ± 0.411	5	nd	nd	3

32	wheat	36.15 ± 0.37	0.003 ± 0.001	nd	-	nd	-	nd	nd	nd	43
33	crop mixture	28.19 ± 0.12	0.687 ± 0.057	33.18 ± 0.41	0.003 ± 0.001	28.05 ± 0.43	1.539 ± 0.497	6	2	3	1
34	crop mixture	31.74 ± 0.19	0.094 ± 0.011	nd	-	nd	-	33	2	13	2
35	wheat	35.8 ± 1.47	0.004 ± 0.003	nd	-	25.13 ± 0.13	11.852 ± 1.045	117	nd	nd	nd
36	wheat	32.88 ± 0.08	0.027 ± 0.001	nd	-	26.62 ± 0.31	4.189 ± 0.877	31	nd	1	0
37	wheat	27.92 ± 0.16	0.794 ± 0.084	33.57 ± 0.3	0.299 ± 0.002	36.99 ± 0.23	0.003 ± <0.001	3	nd	nd	nd
38	wheat	24.63 ± 0.1	7.707 ± 0.51	30.17 ± 0.18	0.019 ± 0.002	nd	-	63	2	nd	5
39	wheat	32.78 ± 0.1	0.026 ± 0.002	nd	-	nd	-	184	nd	nd	nd
40	wheat	29.61 ± 0.06	0.253 ± 0.01	nd	-	nd	-	3	nd	nd	3
41	wheat	36.71 ± 0.44	0.002 ± 0.001	nd	-	25.75 ± 0.31	7.75 ± 1.603	3	nd	nd	4
42	wheat	33.53 ± 0.32	0.018 ± 0.004	nd	-	32.57 ± 0.28	0.061 ± 0.013	2	nd	nd	4
43	wheat	29.83 ± 0.17	0.219 ± 0.026	nd	-	nd	-	1	nd	nd	3
44	wheat	33.84 ± 0.36	0.015 ± 0.004	nd	-	nd	-	8	nd	nd	nd
45	wheat	28.44 ± 0.12	0.47 ± 0.038	nd	-	nd	-	5	2	1	1
46	wheat	36.73 ± 0.47	0.002 ± 0.001	nd	-	nd	-	70	nd	nd	nd
47	wheat	31.73 ± 0.23	0.062 ± 0.009	nd	-	23.49 ± 0.22	38.101 ± 6.06	9	nd	nd	7
48	wheat	31.38 ± 0.19	0.069 ± 0.009	37.18 ± 1.43	0.0401 ± 0.034	27.76 ± 0.19	3.98 ± 0.523	101	nd	nd	nd
49	wheat	35.17 ± 1.98	0.011 ± 0.014	nd	-	28.17 ± 2.42	3.415 ± 3.438	11	nd	4	39
50	crop mixture	31.26 ± 0.2	0.082 ± 0.011	nd	-	32.9 ± 0.16	0.048 ± 0.005	36	nd	nd	nd
51	crop mixture	30.17 ± 0.19	0.172 ± 0.022	nd	-	37.56 ± 0.64	0.002 ± 0.001	3	nd	nd	nd
52	wheat	27.71 ± 0.17	0.9134 ± 0.104	34.2 ± 0.3	0.266 ± 0.001	29.66 ± 0.44	0.497 ± 0.15	21	nd	nd	2
53	wheat	36.08 ± 1.47	0.004 ± 0.003	nd	-	nd	-	nd	nd	nd	5
54	wheat	27.89 ± 0.21	0.811 ± 0.12	34.86 ± 0.3	0.249 ± 0.002	30.66 ± 0.25	0.237 ± 0.044	39	nd	nd	nd
55	wheat	28.59 ± 0.2	0.462 ± 0.064	31.8 ± 0.02	0.006 ± <0.001	29.69 ± 0.24	0.479 ± 0.085	nd	nd	nd	20
56	barley	28.88 ± 0.1	0.593 ± 0.038	34.13 ± 0.42	0.001 ± <0.001	nd	-	5	18	nd	2
57	wheat	31.5 ± 0.13	0.062 ± 0.006	nd	-	nd	-	6	nd	nd	5
58	wheat	28.75 ± 0.14	0.454 ± 0.043	nd	-	nd	-	36	nd	nd	7
59	triticale	21.87 ± 0.1	43.175 ± 3.09	27.46 ± 0.21	0.122 ± 0.017	nd	-	92	9	4	2
60	wheat	33.46 ± <0.01	0.031 ± <0.001	nd	-	23.46 ± 0.07	38.643 ± 1.785	223	nd	nd	8
61	wheat	27.9 ± 0.07	0.68 ± 0.032	32.82 ± 0.03	0.003	nd	-	129	17	36	nd
62	rye	27.67 ± 0.01	0.86 ± 0.005	32.55 ± 0.18	0.004	nd	-	241	nd	nd	nd
63	wheat	28.55 ± 0.15	0.471 ± 0.05	31.13 ± 0.1	0.105 ± 0.016	27.84 ± 0.25	1.757 ± 0.311	24	nd	nd	nd
64	wheat	27.65 ± 0.11	0.948 ± 0.071	32.62 ± 0.3	0.341 ± 0.004	nd	-	54	nd	nd	nd
65	triticale	23.77 ± 0.12	11.722 ± 0.971	30.01 ± 0.07	0.022 ± 0.001	nd	-	5	nd	nd	nd
66	rye	24.63 ± 0.05	6.476 ± 0.201	30.7 ± 0.03	0.014	nd	-	12	nd	nd	nd
67	wheat	32.6 ± 0.04	0.054 ± 0.001	nd	-	nd	-	15	nd	nd	nd
68	wheat	30.45 ± 0.14	0.148 ± 0.014	nd	-	24.22 ± 0.36	23.028 ± 5.924	88	nd	nd	nd
69	wheat	24.7 ± 0.09	7.025 ± 0.431	31.32 ± 0.2	0.231 ± 0.009	27.65 ± 0.23	2.002 ± 0.331	28	nd	nd	nd
70	wheat	30.65 ± 0.14	0.123 ± 0.011	nd	-	30.11 ± 0.02	0.347 ± 0.004	5	6	nd	nd

71	barley	27.54 ± 0.34	1.03 ± 0.237	33.27 ± 0.7		29.71 ± 0.16	0.463 ± 0.053	45	7	16	nd
72	crop mixture	29.87 ± 0.07	0.311 ± 0.014	nd	-	nd	-	4	3	nd	8
73	wheat	35.31 ± 1.01	0.006 ± 0.003	nd	-	30.06 ± 0.23	0.364 ± 0.056	nd	nd	nd	15
74	wheat	26.33 ± .08	2.163 ± 0.119	30.9 ± 0.43	0.012 ± 0.003	nd	-	25	nd	nd	nd
75	triticale	28.35 ± 0.2	0.503 ± 0.068	nd	-	nd	-	31	nd	nd	nd
76	wheat	23.52 ± 0.06	13.924 ± 0.622	28.57 ± 0.17	0.057 ± 0.006	nd	-	17	nd	nd	nd
77	crop mixture	33.51 ± 0.56	0.017 ± 0.007	nd	-	nd	-	38	nd	nd	nd
78	wheat	26.27 ± 0.05	2.254 ± 0.074	31.7 ± 0.13	0.007 ± 0.001	nd	-	244	nd	nd	33
79	wheat	30.74 ± 0.21	0.117 ± 0.017	nd	-	29.38 ± 0.43	0.602 ± 0.174	42	11	20	nd
80	crop mixture	28.21 ± 0.16	0.577 ± 0.061	32.81 ± 0.27	0.003 ± 0.001	nd	-	14	nd	nd	nd
81	wheat	35.77 ± 1.05	0.005 ± 0.003	nd	-	nd	-	6	nd	nd	nd
82	wheat	23.46 ± 0.05	16.146 ± 0.569	30.4 ± 0.19	0.017 ± 0.002	nd	-	182	nd	nd	nd
83	wheat	26.91 ± 0.1	1.572 ± 0.114	30.89 ± 0.12	0.012 ± 0.001	nd	-	194	nd	nd	nd
84	wheat	23.98 ± 0.09	10.838 ± 0.644	30.58 ± 0.1	0.096 ± 0.015	28.52 ± 1.22	1.397 ± 1.277	164	nd	nd	nd
85	wheat	25.76 ± 0.08	3.411 ± 0.177	31.61 ± 0.26	0.007 ± 0.001	nd	-	532	10	9	7
86	wheat	27.17 ± 0.19	1.378 ± 0.171	31.36 ± 0.26	0.009 ± 0.002	nd	-	9	nd	nd	23
87	wheat	32.41 ± 0.19	0.039 ± 0.005	nd	-	31.08 ± 0.25	0.176 ± 0.031	15	nd	nd	12
88	wheat	24.95 ± 0.13	5.514 ± 0.488	29.76 ± 0.2	0.026 ± 0.004	nd	-	88	nd	nd	4
89	wheat	30.79 ± 0.09	0.095 ± 0.006	nd	-	22.47 ± 0.12	78.349 ± 6.508	249	nd	nd	nd
90	wheat	29.00 ± 0.09	0.319 ± 0.02	33.55 ± 0.25	0.002 ± <0.001	nd	-	35	nd	nd	nd
91	wheat	30.71 ± 0.17	0.121 ± 0.014	nd	-	nd	-	650	nd	nd	nd
92	wheat	31.59 ± 0.47	0.057 ± 0.02	nd	-	28.9 ± 2.22	1.837 ± 1.859	16	nd	nd	nd
93	wheat	35.81 ± 0.89	0.004 ± 0.003	nd	-	nd	-	57	nd	nd	nd
94	wheat	29.57 ± 0.07	0.237 ± 0.012	35.87 ± 1.62	0.107 ± 0.074	26.09 ± 0.05	12.631 ± 0.461	463	nd	nd	nd
95	wheat	25.8 ± 0.18	3.175 ± 0.397	32.73 ± 0.34	0.696 ± 0.161	24.02 ± 0.13	52.888 ± 4.588	487	nd	nd	nd
96	wheat	33.19 ± 0.23	0.023 ± 0.004	nd	-	nd	-	58	nd	nd	nd
97	wheat	35.16 ± <0.01	0.005 ± <0.001	nd	-	23.86 ± 0.31	29.59 ± 6.754	352	nd	nd	nd
98	wheat	34.74 ± 1.08	0.007 ± 0.004	nd	-	nd	-	89	nd	nd	nd
99	wheat	27.61 ± 0.06	0.915 ± 0.037	33.79 ± 0.15	0.331 ± 0.035	22.22 ± 0.1	184.408 ± 12.905	1014	nd	nd	nd
100	wheat	34.0 ± 1.03	0.015 ± 0.008	nd	-	35.84 ± 0.22	0.006 ± 0.001	15	nd	nd	nd
101	wheat	34.49 ± 0.88	0.01 ± 0.006	nd	-	nd	-	39	nd	nd	nd
102	wheat	27.62 ± 0.07	0.904 ± 0.043	34.35 ± 0.25	0.227 ± 0.04	22.27 ± 0.08	177.73 ± 10.35	928	nd	nd	nd
103	wheat	35.58 ± 0.99	0.004 ± 0.003	nd	-	nd	-	19	nd	nd	nd
104	wheat	24.49 ± 0.24	7.823 ± 1.233	35.3 ± 0.7	0.125 ± 0.052	27.11 ± 0.18	6.231 ± 0.778	220	nd	nd	nd
105	wheat	34.85 ± 1.18	0.007 ± 0.005	nd	-	nd	-	19	nd	nd	13
106	wheat	36.46 ± 0.95	0.002 ± 0.001	nd	-	nd	-	31	nd	nd	nd
107	unknown	25.92 ± 0.06	2.811 ± 0.117	32.01 ± 0.35	0.006 ± 0.001	29.32 ± 0.53	0.636 ± 0.253	19	nd	nd	14
108	unknown	27.94 ± 0.03	0.661 ± 0.015	33.345 ± 0.21	0.002 ± <0.001	nd	-	31	nd	nd	4

References

1 Baturo-Cieśniewska, A.; Suchorzyńska, M. Verification of the effectiveness of SCAR (sequence characterized amplified region) primers for the identification of Polish strains of *Fusarium culmorum* and their potential ability to produce B-trichothecenes and zearalenone. *Int. J. Food Microbiol.* **2001**, *148*, 168–176, doi:10.1016/j.ijfoodmicro.2011.05.017.

2 Kulik, T.; Ostrowska, A.; Buśko, M.; Pasquali, M.; Beyer, M.; Stenglein, S.; Załuski, D.; Sawicki, J.; Treder, K.; Perkowski, J. Development of an FgMito assay: A highly sensitive mitochondrial based qPCR assay for quantification of *Fusarium graminearum* sensu stricto. *Int. J. Food Microbiol.* **2015**, *210*, 16–23, doi:10.1016/j.ijfoodmicro.2015.06.012.

3 Waalwijk, C.; van der Heide, R.; de Vries, I.; van der Lee, T.; Schoen, C.; Corainville, G.C.; Häuser-Hahn, I.; Kastelein, P.; Köhl, J.; Lonnet, P.; et al. Quantitative detection of *Fusarium* species in wheat using TaqMan. *Eur. J. Plant Pathol.* **2004**, *110*, 481–494, doi:10.1023/B:EJPP.0000032387.52385.13.
